# Incidence and risk factors of surgical site infections and related antibiotic resistance in Freetown, Sierra Leone: a prospective cohort study

**DOI:** 10.1186/s13756-022-01078-y

**Published:** 2022-02-21

**Authors:** Sulaiman Lakoh, Le Yi, Stephen Sevalie, Xuejun Guo, Olukemi Adekanmbi, Isaac O. Smalle, Nathaniel Williams, Umu Barrie, Celesis Koroma, Yongkun Zhao, Matilda N. Kamara, Constance Cummings-John, Darlinda F. Jiba, Enanga Sonia Namanaga, Betsy Deen, Juling Zhang, Anna Maruta, Christiana Kallon, Peng Liu, Haja Ramatulai Wurie, Joseph Sam Kanu, Gibrilla F. Deen, Mohamed Samai, Foday Sahr, Emmanuel Firima

**Affiliations:** 1 College of Medicine and Allied Health Sciences, University of Sierra Leone, Freetown, Sierra Leone; 2 Ministry of Health and Sanitation, Government of Sierra Leone, Freetown, Sierra Leone; 3 Changchun Veterinary Research Institute, Chinese Academy of Agricultural Sciences, Changchun, 130000 China; 4 34 Military Hospital, Freetown, Sierra Leone; 5 Department of Medicine, College of Medicine, University of Ibadan, Ibadan, Nigeria; 6 Department of Medicine, University College Hospital, Ibadan, Nigeria; 7 Infectious Disease Research Network, Freetown, Sierra Leone; 8 Department of Clinical Laboratory, The Fifth Medical Center of Chinese PLA General Hospital, Beijing, 100039 China; 9 World Health Organization Country Office, Freetown, Sierra Leone; 10 Department of Emergency Medicine, The Fifth Medical Center of Chinese PLA General Hospital, Beijing, 100039 China; 11 Clinical Research Unit, Department of Medicine, Swiss Tropical and Public Health Institute, Basel, Switzerland; 12 University of Basel, Basel, Switzerland; 13 SolidarMed, Butha-Buthe, Lesotho; 14 Centre for Multidisciplinary Research and Innovation, Abuja, Nigeria

**Keywords:** Surgical site infections, *Enterobacteriaceae*, Sierra Leone, Antibiotic resistance, Extended spectrum beta-lactamase

## Abstract

**Background:**

There is limited information on surgical site infections (SSI) and the related antibiotic resistance needed to guide their management and prevention in Sierra Leone. In this study, we aimed to establish the incidence and risk factors of SSI and the related antibiotic resistance among adults attending a tertiary hospital, and a secondary health facility in Freetown, Sierra Leone.

**Methods:**

This is a prospective cohort study designed to collect data from adult (18 years or older) patients who attended elective and emergency surgeries at two hospitals in Freetown between February and July, 2021. Data analysis was done using STATA version 16.

**Results:**

Of 338 patients, 245 (72.5%) and 93 (27.5%) had their surgeries at the tertiary and secondary hospitals, respectively. Many were males 192 (56.8%), less than 35 years 164 (48.5%), and 39 (11.5%) developed an SSI. Of the 39 patients who acquired an SSI, 7 (17.9%) and 32 (82.1%) had their surgeries at the secondary and tertiary hospitals, respectively. The incidence of SSI is higher in contaminated 17 (43.6%) than in clean-contaminated 12 (30.8%) and clean 10 (25.6%) wounds. Wound swabs were collected in 29 (74.4%) patients, of which 18 (62.1%) had bacterial growth. In total, 49 isolates of 14 different bacteria including gram-negative 41 (83.7%) and gram-positive 8 (16.3%) isolates were identified. Of these, 32 (65.3%) were *Enterobacteriaceae*, 9 (18.4%) were Non-fermenting gram-negative bacilli and 10 (12.2%) were *Enterococci*. The most common isolates were *Escherichia coli* (12, 24.5%), *Klebsiella pneumoniae* (10, 20.4%), *Acinetobacter baumannii (*5, 10.2%), *Klebsiella oxytoca* (4, 8.2%) *and Enterococcus faecalis* (4, 8.2%). The *Enterobacteriaceae* were either resistance to carbapenems (4, 8.2%) or were extended-spectrum beta-lactamase (ESBL) producing organisms (29, 59.2%). Male sex [*p* = 0.031], an ASA score ≥ 2 [*p* = 0.020), administration of general anaesthesia [*p* = 0.018] and elevated fasting glucose [*p* = 0.033] were predictive of SSI.

**Conclusion:**

The incidence of SSI in this study is comparable to other low- and middle-income countries, but a substantial proportion of these postoperative wounds have an ESBL-producing *Enterobacteriaceae*. Therefore, routine surveillance of SSI and related antibiotic resistance is required in resource-limited settings.

## Introduction

Surgical site infections (SSI) are a major public health threat to the success of global surgery, especially in developing countries. Although there is no comprehensive global data, the prevalence of SSI in low- and middle-income countries (LMICs) is higher than that in high-income countries (HICs) [1]. According to estimates by the World Health Organization (WHO) in 2011, the incidence of SSI in HICs ranges from 1.2 to 5.2%, while that in LMICs is 10.8% [2]. Chu reports that the incidence of SSI in HICs is 9.4%, while that in LMICs is 23.2% [3]. In sub-Saharan Africa, the prevalence of SSI is 16%, ranging from 6.8 to 26% [4].

The high burden of SSI in LMICs clearly demonstrates the need to strengthen global support for low-income countries to improve surgical outcomes. Global initiatives to improve surgical outcomes have been developed for use, especially for LMICs. The WHO guidelines for the use of the WHO surgical safety checklist to prevent SSI are one of the global quality initiatives to improve surgical outcomes in under-resourced settings [5]. Although the use of the checklist has expanded globally, the results of a multi-country collaborative study show that it is ineffective in improving surgical outcomes if local preventive measures are not followed [6]. Hence, a more comprehensive approach is needed to prevent and control SSI.

The adverse effects of SSI on the health system and patients and their caregivers further prove the necessity of prevention, as they increase healthcare costs, lengthen hospital stays, and increase postoperative morbidity and mortality [7]. Complicating the problem is the intertwined relationship between SSI and the increasing burden of antimicrobial resistance, which is exacerbated by the inappropriate use of antibiotics in surgical prophylaxis and poor infection prevention and control practices [8]. Despite these huge challenges in preventing SSI and antibiotic resistance, there are limited data on SSI-related antibiotic resistance in many countries in sub-Saharan Africa.

In response to the 2014/2016 ebola epidemic in Sierra Leone, a National Infection Prevention and Control Policy that includes surveillance and control of SSIs and other HAIs was developed [9]. Yet, there is limited information in the literature on the incidence and risk factors of SSI and the related antibiotic resistance needed to guide their prevention and optimal management. Previous studies conducted at hospitals in Sierra Leone recorded an SSI prevalence rates between 7.3 and 10.9% in caesarean section wounds [10, 11]. However, the relevance of these studies is limited to describing the prevalence and risk factors of SSI at the level of maternal care. There are no data on postoperative wound isolates and their antibiotic resistance. It is worthwhile, therefore, to provide more comprehensive data on surgical site infections in Sierra Leone to inform public health policies and practices.

In this study, we aimed to establish the incidence and risk factors of SSIs and assess the related antibiotic resistance among adults attending an urban tertiary hospital, and a secondary health facility in Freetown to inform interventions on the prevention and control of SSIs in Sierra Leone and similar countries.

## Methods

### Study design

This is an observational prospective cohort study designed to collect data from patients participating in elective and emergency surgeries.

### Study setting

The study was conducted at an urban tertiary hospital and secondary health facility in Freetown, Sierra Leone. The tertiary hospital was established to conduct research and provides training and clinical services. In total, the hospital has a capacity of 300 beds. Five of the 10 wards of the hospital admit surgical cases including those operated in the two surgical theatres. An average of 20 adult patients a week undergoes surgeries in this hospital, of which about 16 are elective cases. Unlike the tertiary hospital which provides surgical services to only non-pregnant patients, the secondary hospital provides both surgical and obstetric services. It has a total of 42 beds and performs an average of 4 surgeries a week.

### Sample size

We used a standard sample size calculation with an α of 0.05, and a power of 0.8 to obtain a minimum sample size of 224 surgical case. In total, however, 245 and 93 post-operative patients were recruited at the tertiary and secondary hospitals, respectively.

### Sampling, clinical procedure and definitions

Adult (18 years or older) patients who had both elective and emergency surgeries at the selected tertiary and secondary hospitals were sequentially enrolled in the study from February 09, 2021 to July 31, 2021 (estimated at 22 weeks). The eligibility criteria for inclusion in the study were patients who participated in either emergency or elective surgeries and were 18 years or older. Patients who refused consent, had amputations or other bone surgeries and patients whose infected wounds were not classified as SSI by definition (patients with suture abscesses, local puncture wounds, trauma-related wounds, episiotomy or infected burn wounds) were excluded from the study.

Two trained nurses (one in each hospital) collected the data on the assessment of surgical site infections, and their risk factors from the patients’ files as recorded by the managing teams, and by patients/ward nurses’ interviews.

Patients were first recruited in the preoperative/perioperative ward of the hospital to obtain consent and detailed information on their socio-demographic characteristics and preoperative preparation. After the surgery, patients were followed-up at the post-operative wards to obtain documented intraoperative information on their records. During routine wound cleaning, weekly follow-up evaluations were performed in the minor operating room of the hospitals to evaluate the wound status and physiological parameters. Follow-up evaluation continued until the wound had healed or 30 days after surgery, which is usually the prescribed period for the development of SSI without implants [12]. Patients who may have decided to obtain post-operative services at other institutions or request that their care be transferred to other institutions to continue their postoperative care were followed up directly by phone or through their service providers.

Clinically, a wound was identified as an SSI if it occurred within 30 days post-surgery and had at least a purulent discharge from the incision, local swelling, redness, pain or tenderness, wound abscess, foul smell, or fever [13]. Wound swabs were collected from the patients with an SSI and sent to the laboratory for bacterial culture and Antibiotic Susceptibility Testing (AST).

### Laboratory procedure

Upon reaching the laboratory, wound swab samples were streaked onto the chromogenic agar plate (CHROMagar^™^ orientation) and incubated aerobically at 37 °C for 18 to 24 h. Where there was a bacterial growth, a colony was picked and streaked onto a Brain Heart Infusion (BHI) agar plate for purification and Gram staining. All isolates were cultured twice to ensure they were pure but there were no discordant results.

A VITEK 2 compact system (bioMérieux, France) was used for identification and antibiotic susceptibility testing of isolates from pure cultures. Using a DensiCHEK Plus turbidimeter (bioMérieux, France), a solution of bacteria in saline was prepared in polystyrene tubes (bioMérieux, France) to levels between 0.5 and 0.63 MacFarland standard. Antibiotic susceptibility tests were performed by adding 145 μl (for gram-negative bacteria) or 280 μl (for gram-positive bacteria) of suspension into a new polystyrene tube as per the manufacturer’s instructions.

The isolates suspensions were loaded on the VITEK 2 compact system and incubated overnight at 37 °C. All the culture and antibiotic susceptibility results generated were printed and dispatched to the research team.

### Definition of termsof terms [12, 13]

#### Wound classification based on the potential of infection

- Clean wound is an uninfected operative wound in which no inflammation is encountered and the respiratory, alimentary, genital or uninfected urinary tracts are not entered
- Clean-contaminated wound is an operative wound in which the respiratory, alimentary, genital or urinary tracts are entered under controlled conditions and without unusual contamination.
- Contaminated wound is an open, fresh, accidental wound.
- Dirty wound is an old traumatic wound with retained devitalized tissue or wound involving existing clinical infection or perforated viscera.

#### Wound classification based on the extent of involvement

- Superficial surgical site infection confines to the skin and subcutaneous tissue and it is characterised by the presence of either purulent drainage with or without laboratory confirmation or microbial isolates from superficial incision or the presence of signs and symptoms of infection at the site, or diagnosis of SSI by clinician.
- Deep surgical site infections is the presence of at least purulent drainage from the deep incision, spontaneous dehisces or a deliberately opened deep incision in a patient with at least one of the following signs or symptoms: fever (> 38 °C), localized pain, or tenderness, unless site is culture-negative, an abscess or other evidence of infection involving the deep incision soft tissues or diagnosis of a deep incisional SSI by the attending clinician.
- Organ/space SSI is an infection which involves any part of the anatomy other than the incision, which was opened or manipulated during an operation and at least purulent drainage from a drain that is placed through a stab wound into the organ/space, isolate from an aseptically obtained culture of fluid or tissue in the organ/space, abscess or other evidence of infection involving the organ/space that is found on direct examination, during re-operation, or by histopathologic or radiologic examination, and diagnosis of an organ/space SSI by a surgeon or attending physician.

### Data collection

The data was collected using a password-protected Epidata platform. On the preoperative/perioperative wards, socio-demographic variables and data on the underlying surgical condition, duration of hospitalization before surgery, body mass index, comorbidities, and preoperative skin preparations were entered into the Epidata software. The employment status of the study participants were categorized into unemployed, retired, student, informal sector (artisans, traders, farmers, fishing etc.) and formal sector (civil servants, teachers, police, military etc.).

The American Society of Anesthesiologists’ (ASA) preoperative assessment score was evaluated for each patient according to preoperative physical status [14].

Information on intraoperative surgical interventions was collected on the post-operative wards. Further assessment of the risk factors for surgical site infections involved the collection of data on antibiotic use post-surgery, the duration of postoperative admission, use of central and peripheral vascular lines, urinary catheters, nasogastric tubes, and duration of surgical drains.

The bacterial culture data were recorded at the end of the admission.

### Data management and analysis

Data were exported from Epi-Data platform into Microsoft Excel, cleaned, coded and transferred into STATA version 16 (StataCorp LLC) for analysis. Descriptive statistics such as frequencies and percentages were used to present demographic and clinical characteristics of the study participants. Univariate and multivariable logistic regression were used to identify factors associated with surgical site infections (SSIs). Variables which had a *p* value < 0.25 in the univariate logistic regression model were fitted to the multivariable logistic regression model to identify predictors of SSIs. Findings were presented as crude odds ratio after univariate analysis, and adjusted odds ratio (aOR) following multivariable analysis, with their 95% confidence intervals (CI). A *p* value of less than 0.05 was considered statistically significant.

### Ethical consideration

Ethical approval was obtained from the Sierra Leone Ethics and Scientific Review Committee of the Ministry of Health and Sanitation. Prior to the start of the study, permission was obtained from the administrations of the hospitals and heads of departments.

A participant information sheet was provided to all patients prior to their recruitment or was read aloud for those unable to read. Informed written consent was taken with signatures or thumbprints (for participants unable to read).

Participants who refused to consent were still offered appropriate management or advice but their details were not recorded for the purposes of the study. Participants were free to withdraw at any time, without giving a reason up until the completion of the study. Swab cultures and blood glucose checks requested for the purpose of the research were at no cost to participants.

## Results

### Demographic characteristics of study participants

A total of 338 patients were followed up for 30 days after surgery. Of this, 245 (72.5%) and 93 (27.5%) had surgery at the tertiary and secondary hospitals, respectively.

The majority were males 192 (56.8%), less than 35 years 164 (48.5%), and married 208 (61.5%). The highest educational attainment of the patients was secondary level 155 (46%). Many patients worked in the informal sector 175 (51.8%), never took alcohol 295 (87.3%), or smoked cigarettes 293 (86.7%). Other details of the patients’ socio-demographic characteristics are presented in Table 1.

**Table 1:** Socio-demographic characteristics of the study participants (N = 338)

Variables	Frequency	%
Hospital		
Secondary hospital	93	27.5
Tertiary hospital	245	72.5
Sex		
Female	146	43.2
Male	192	56.8
Age		
< 35	164	48.5
35–60	147	43.5
> 60	27	8.0
Marital status		
Single	107	31.7
Married	208	61.5
Divorced/separated/widowed	23	6.8
Level of education		
None	87	25.8
Primary	26	7.7
Secondary	155	46.0
Tertiary	69	20.5
Occupation		
Unemployed	16	4.7
Student	51	15.1
Informal sector	175	51.8
Formal sector	87	25.7
Retired	9	2.7
Alcohol consumption		
No	295	87.3
Yes	43	12.7
Cigarette smoking		
No	293	86.7
Yes	45	13.3
Other substance use		
No	332	98.2
Yes	6	1.8

### Details of the clinical characteristics and surgical management of the study participants

Most 313 (92.6%) patients had a body mass index (BMI) below 25 kg/m^2^, and 89 (26.3%) patients had comorbidities. Preoperatively, 134 (39.8%) patients were admitted to the hospital for more than 24 h and received antibiotic therapy 90 (26.6%) and blood transfusion 25 (7.4%). Only 64 (18.9%) patients had an ASA score of 2 and above. Most 213 (63%) surgeries were abdominal. Forty patients (11.8%) had elevated fasting blood glucose levels of 5.6 mmol/l or higher (Table 2).

**Table 2:** Clinical characteristics of study participants (N = 338)

Variables	Frequency	%
ASA score		
< 2	274	81.1
≥ 2	64	18.9
Surgical site		
Non abdominal	63	18.6
Intra-abdominal	213	63.0
Obstetric	62	18.3
BMI		
< 25	313	92.6
≥ 25	25	7.4
Pre-op blood transfusion		
No	312	92.6
Yes	25	7.4
Comorbidities		
Absent	249	73.7
Present	89	26.3
Pre-op admission		
≤ 24 h	203	60.2
> 24 h	134	39.8
Pre-op antibiotics		
No	248	73.4
Yes	90	26.6
Surgery duration		
< 120 min	293	86.7
≥ 120 min	45	13.3
Type of anaesthesia		
Local/spinal	157	46.5
General	181	53.5
Type of suture		
Absorbable only	137	40.5
Non-absorbable only	83	24.6
Both	12	3.6
Not reported	106	31.4
Intra-op blood transfusion		
No	293	86.9
Yes	44	13.1
Number of surgeons		
≤ 2	265	78.4
> 2	73	21.6
Number of scrub nurses		
1	325	96.1
> 1	13	3.9
Number of anaesthetists		
1	219	64.8
> 1	119	35.2
Intra-op antibiotics		
No	104	30.9
Yes	233	69.1
Type of wound		
Clean	191	56.5
Clean-contaminated	116	34.3
Contaminated	31	9.2
Post op antibiotics		
No	55	16.3
Yes	282	83.7
Post op NG tube		
No	296	88.1
Yes	40	11.9
Post op urinary catheter		
No	199	58.9
Yes	139	41.1
Surgical drains use		
No	266	78.9
Yes	71	21.1
Impaired glucose tolerance		
No	298	88.2
Yes	40	11.8
Surgical site infection (SSI)		
Absent	299	88.5
Present	39	11.5
Type of SSI		
Superficial	27	69.0
Deep	12	31.0

All the patients underwent some form of skin preparation before surgery. Among them, 295 (87.3%) had non-antiseptic pre-operative shower, 2 (0.6%) had antiseptic pre-operative shower, and 314 (92.9%) had hair removal by shaving.

In 157 (46.5%) cases, patients received either local or spinal anesthesia, and in 45 (13.3%) cases, the operation lasted at least 2 h. The number of surgeries attended by more than 2 surgeons was 73 (21.6%), whereas 13 (3.9%) and 119 (35.2%) surgeries, respectively were attended by more than one scrub nurse or anesthetist. Most surgical wounds were clean 191 (56.5%). During surgery, antibiotics were administered to 233 (69.1%) patients, and the number of patients with a blood transfusion increased to 44 (13.1%). Although the type of suture material used was not reported in 106 (31.4%) cases, in 137 (40.5%) cases, absorbable sutures were used for wound closure.

Postoperatively, 71 (21.1%) patients had at least one surgical drainage tube placed; 40 (11.9%) and 139 (41.1%) patients had nasogastric and urinary catheters placed in situ, respectively. Antibiotics were given to 282 (83.7%) patients. See Table 2 for details.

### Incidence and characteristics of surgical site infections

Overall, 39 (11.5%) patients developed a surgical site infection (SSI). Of the 39 cases of SSI, 27 (69%) were deep, while 12 (31%) were superficial. Of the 39 patients who developed SSI, 7 (17.9%) were from secondary hospital, while 32 (82.1%) had their treatment in the tertiary hospital. The incidence of SSI is higher in contaminated 17 (43.6%) than in clean-contaminated 12 (30.8%) and clean 10 (25.6%) wounds (Tables 2 and 3).

**Table 3:** Univariate and multivariable logistic regression of factors associated with surgical site infection

Variables	Surgical site infection		Crude odds ratio (confidence interval)	*p* value	Adjusted odds ratio (confidence interval)	*p* value
	Yes N (%) 39 (11.5)	No N (%) 299 (88.5)				
Hospital study site						
Secondary	7 (17.9)	86 (28.8)	1		1	
Tertiary	32 (82.1)	213 (71.2)	1.85 (0.78–4.34)	0.160	4.8 (0.62–36.91)	0.132
Sex						
Female	13 (33.3)	133 (44.5)	1		1	
Male	26 (66.7)	166 (55.5)	1.6 (0.79–3.24)	0.189	3.9 (1.13–13.33)	**0.031**
Age						
< 35	19 (48.7)	145 (48.5)	1			
35–60	16 (41.0)	131 (43.8)	0.93 (0.46–1.89)	0.845		
> 60	4 (10.3)	23 (7.7)	1.33 (0.41–4.25)	0.634		
Marital status						
Single	12 (30.8)	95 (31.8)	1			
Married	26 (66.7)	182 (60.9)	1.13 (0.55–2.34)	0.740		
Divorced/separated/widowed	1 (2.5)	22 (7.3)	0.36 (0.04–2.91)	0.338		
Alcohol consumption						
No	33 (84.6)	262 (87.6)	1			
Yes	6 (15.4)	37 (12.4)	1.29 (0.51–3.28)	0.597		
Cigarette smoking						
No	33 (84.6)	260 (87.0)	1			
Yes	6 (15.4)	39 (13.0)	1.21 (0.48–3.08)	0.689		
ASA						
< 2	15 (38.5)	259 (86.6)	1		1	
≥ 2	24 (61.5)	40 (13.4)	10.4 (5.01–21.41)	**< 0.001**	4.1 (1.26–13.50)	**0.020**
BMI						
< 25	37 (94.9)	276 (92.3)	1			
≥ 25	2 (5.1)	23 (7.7)	0.65 (0.15–2.86)	0.568		
Site of surgery						
Non abdominal	4 (10.3)	59 (19.7)	1		1	
Intra-abdominal	31 (79.5)	182 (60.9)	2.51 (0.85–7.41)	0.095	1.4 (0.33–5.90)	0.659
Caesarian sections	4 (10.3)	58 (19.4)	1.02 (0.24–4.26)	0.981	2.0 (0.14–30.05)	0.608
Pre-op blood transfusion						
No	33 (84.6)	279 (93.6)	1		1	
Yes	6 (15.4)	19 (6.4)	2.67 (0.99–7.16)	0.051	0.3 (0.06–1.30)	0.105
Comorbidities						
Absent	27 (69.2)	222 (74.3)	1			
Present	12 (30.8)	77 (25.7)	1.28 (0.62–2.65)	0.504		
Pre-op admission						
≤ 24 h	14 (35.9)	189 (63.4)	1		1	
> 24 h	25 (64.1)	109 (36.6)	3.1 (1.54–6.21)	**0.001**	4.1 (1.47–11.66)	**0.007**
Pre-op antibiotics						
No	16 (41)	232 (77.6)	1		1	
Yes	23 (59)	67 (22.4)	5.0 (2.49–9.96)	**< 0.001**	1.2 (0.43–3.52)	0.700
Surgery duration						
< 120 min	29 (74.4)	264 (88.3)	1		1	
≥ 120 min	10 (25.6)	35 (11.7)	2.6 (1.17–5.79)	**0.019**	1.2 (0.38–3.85)	0.738
Type of anaesthesia						
Local/spinal	6 (15.4)	151 (50.5)	1		1	
General	33 (84.6)	148 (49.5)	5.6 (2.28–13.79)	**< 0.001**	6.8 (1.40–33.08)	**0.018**
Intra-op blood transfusion						
No	23 (59)	270 (90.6)	1		1	
Yes	16 (41)	28 (9.4)	6.7 (3.18–14.16)	**< 0.001**	2.1 (0.58–7.55)	0.263
Number of surgeons						
≤ 2	24 (61.5)	241 (80.6)	1		1	
> 2	15 (38.5)	58 (19.4)	2.6 (1.28–5.26)	**0.008**	0.3 (0.09–0.97)	**0.045**
Number of scrub nurses						
1	37 (94.9)	288 (96.3)	1			
> 1	2 (5.1)	11 (3.7)	1.4 (0.30–6.63)	0.660		
Number of anaesthetists						
1	22 (56.4)	197 (65.9)	1		1	
> 1	17 (43.6)	102 (34.1)	1.5 (0.76–2.93)	0.246	1.3 (0.51–3.41)	0.565
Intra-op antibiotics						
No	7 (17.9)	97 (32.5)	1		1	
Yes	32 (82.1)	201 (67.5)	2.2 (0.94–5.18)	0.069	0.8 (0.22–3.24)	0.806
Type of wound						
Clean	10 (25.6)	181 (60.5)	1		1	
Clean-contaminated	12 (30.8)	104 (34.8)	2.1 (0.87–5.00)	0.098	0.8 (0.19–3.52)	
Contaminated	17 (43.6)	14 (4.7)	22.0 (8.49–56.93)	**< 0.001**	6.5 (1.04–40.20)	**0.045**
Post-op antibiotics						
No	3 (7.7)	52 (17.5)	1		1	
Yes	36 (92.3)	246 (82.5)	2.5 (0.75–8.55)	0.133	0.4 (0.09–2.07)	0.289
Post-op NG tube						
No	24 (63.2)	272 (91.3)	1		1	
Yes	14 (36.8)	26 (8.7)	6.1 (2.82–13.21)	**< 0.001**	0.7 (0.19–2.62)	0.610
Urinary catheter						
No	11 (28.2)	188 (62.9)	1		1	
Yes	28 (71.8)	111 (37.1)	4.3 (2.07–9.00)	**< 0.001**	3.9 (0.98–15.20)	0.053
Surgical drains use						
No	16 (41)	250 (83.9)	1			
Yes	23 (59)	48 (16.1)	7.5 (3.69–15.21)	**< 0.001**	0.5 (0.10–3.00)	0.485
Impaired glucose tolerance						
No	30 (76.9)	268 (89.6)	1		1	
Yes	9 (23.1)	31 (10.4)	2.6 (1.13–5.96)	**0.025**	3.5 (1.10–10.91)	**0.033**

Bold values indicate the statistically significant

### Factors associated with surgical site infection

Following univariate logistic regression, ASA score of ≥ 2 (*p* < 0.001), pre-op admission of more than 24 h (*p* = 0.001), administration of antibiotics pre-operatively (*p* < 0.001), duration of surgery ≥ 2 h (*p* = 0.019), administration of general anaesthesia (*p* < 0.001), intraoperative blood transfusion (*p* < 0.001), having more than 2 surgeons participating in a surgery (*p* = 0.008), contaminated wound (*p* < 0.001), elevated fasting glucose levels ≥ 5.6 mmol/l (*p* = 0.025), and placement of postoperative nasogastric tube (*p* < 0.001), urinary catheter (*p* < 0.001) and surgical drain (*p* < 0.001) were statistically associated with development of a surgical site infection. Other factors with *p* < 0.25 were sex, site of surgery, preoperative blood transfusion, hospital study site, number of anaesthetists at surgery, intraoperative antibiotics as well as use of antibiotics postoperatively (Table 3).

Multivariable logistic regression showed that male patients [aOR 3.9, 95% CI (1.13–13.33); *p* = 0.031] and patients with an ASA score of ≥ 2 [aOR 4.1, 95% CI (1.26–13.50); *p* = 0.020) were four times more likely to develop SSI. Contaminated surgical wounds were over six times more likely to become infected compared to clean wounds, [aOR 6.5, 95% CI (1.04–40.20); *p* = 0.045]. Being on admission for more than 24 h prior to surgery [aOR 4.1, 95% CI (1.47–11.66); *p* = 0.007], elevated fasting glucose was a statistically significant predictor of surgical site infection, [aOR 3.5, 95% CI (1.10–10.91); *p* = 0.033] and use of general anaesthesia [aOR 6.8, 95% CI (1.40–33.08); *p* = 0.018], were predictive of SSI. When more than two surgeons attended the surgery, patients were 70% less likely to have infected wounds, [aOR 0.30, 95% CI (0.09–0.97); *p* = 0.045]. See Table 4 for details (Table 3).

**Table 4:** Bacterial isolates and patterns of antibiotic resistance of isolates from post-operative wounds

Bacterial isolates	N = 49	%	Resistant patterns of isolates	N	%
Enterobacteriacea	32	65.3	Enterobacteriacea	32	65.3
*Escherichia coli*	12	24.5	Extended-spectrum beta-lactamase (ESBL) producing organisms	29	59.2
*Klebsiella pneumoniae ssp pneumonia*	10	20.4	Carbapenem resistance *Enterobacteriacea*	4	8.2
*Klebsiella oxytoca*	4	8.2			
*Proteus mirabilis*	3	6.2			
*Morganella morganii ssp morganii*	1	2.0			
*Providencia stuartii*	1	2.0			
*Serratia fonticola*	1	2.0			
Non-fermenters	9	18.4	**Non-fermenters**		
*Acinetobacter baumannii complex*	5	10.2	Carbapenem-resistant *A. baumanii* (CRAB)	0	0.0
*Pseudomonas aeruginosa*	3	6.2	Carbapenem-resistant *P. aeruginosa* (CRPA)	0	0.0
*Pseudomonas luteola*	1	2.0		0	0.0
Gram positive cocci	8	16.3	Vancomycin resistance *Enterococci*		
*Enterococcus faecalis*	5	10.2			
*Enterococcus raffinosus*	1	2.0			
*Staphylococcus haemolyticus*	1	2.0			
*Kocuria kristinae*	1	2.0			

### Profile of bacterial isolates and antibiotics resistance patterns

Of patients who developed an SSI, wound swabs were collected in 29 (74.4%) patients and 18 (62.1%) had bacterial growth. In total, 49 isolates of 14 different bacteria including both gram negative (41, 83.7%) and gram positive (8, 16.3%) isolates were identified. Of these, 32 (65.3%) are *Enterobacteriaceae*, 9 (18.4%) were Non-fermenting gram-negative bacilli, and 8 (16.3%) are gram-positive cocci. The most common isolates are *Escherichia coli* 12 (24.5%), *Klebsiella pneumoniae spp pneumonia* 10 (20.4%), *Acinetobacter baumannii complex* 5 (10.2%), *Klebsiella oxytoca* (4, 8.2%) *and Enterococcus faecalis* (4, 8.2%). The *Enterobacteriaceae* were either resistance to carbapenems 4 (8.2%) or were extended-spectrum beta-lactamase (ESBL) producing organisms 29 (59.2%). There were no carbapenem-resistant non-fermenting gram negative bacilli (Table 4).

Most of the gram negative bacteria isolates are resistant to quinolones, penicillins, some aminoglycosides and second, third and fourth generation cephalosporin. Susceptibility to piperacillin/tazobactam, amikacin and carbapenems was recorded for many gram negative pathogens (Table 5). Again, all the *Enterococci* and *S. haemolyticus* have good susceptibility to tegecycline, vancomycin and linezolid (Table 6).

**Table 5:** Antibiotic resistance profile of Gram-negative bacteria isolates

Antibiotics	Resistance rate (%)^®^						
	*E. coli* *N* = 12	*K. pneumoniae N* = 10	*Pseudomonas spp.* *N* = 4	*A. baumanii* *N* = 5	*K. oxytoca* *N* = 4	*P. mirabilis* *N* = 3	*M. morgagni* *N* = 1
Imipenem	0.0	0.0	0.0	0.0	25.0	0.0	100.0
Meropenem	0.0	0.0	0.0	–	25.0	0.0	0.0
Ampicillin	81.8	100.0	–	80.0	–	–	–
Ampicillin-sulbactam	75.0	81.8	–	20.0	–	–	–
Ciprofloxacin	100.0	81.8	75.0	36.0	50.0	100.0	0.0
Levofloxacin	100.0	54.5	75.0	40.0	50.0	0.0	0.0
Amikacin	0.0	9.1	50.0	17.0	25.0	100.0	0.0
Gentamycin	68.0	63.0	75.0	56.0	25.0	100.0	0.0
Tobramycin	50.0	72.0	75.0	29.0	75.0	100.0	0.0
Aztreonam	75.0	63.0	–	–	50.0	–	–
Co-trimoxazole	83.3	72.0	–	93.0	75.0	–	0.0
Nitrofurantoin	36.4	13.3	–	84.0	0.0	–	0.0
Ceftriaxone	100.0	90.9	–	36.0	75.0	–	0.0
Cefuroxime	100.0	100.0	–	100.0	100.0	–	100.0
Cefuroxime Axetil	100.0	100.0	–	89.0	75.0	–	100.0
Cefotetan	25.0	0.0	–	69.0	100.0	–	0.0
Cefazolin	81.8	81.8	–	92.0	100.0	–	100.0
Ceftazidime	100.0	100.0	0.0	46.0	100.0	0.0	0.0
Cefepime	81.8	100.0	25.0	29.0	75.0	0.0	0.0
Piperacillin/tazobactam	16.7	9.1	0.0	0.0	25.0	0.0	0.0

^**®**^Percentages = number of resistant antibiotics/total number of antibiotics tested

(–) indicate not tested

**Table 6:** Antibiotic resistance pattern of Gram-positive bacteria

	Resistance rate (%)^®^		
	*E. faecalis* (N = 4)	*E. raffinosus* (N = 1)	*S. haemolyticus* (N = 1)
Benzylpenicillin	44.0	100.0	100.0
Oxacillin	–	–	100.0
Ampicillin	60.0	100.0	0.0
Ampicillin-sulbactam	28.0	100.0	0.0
Erythromycin	50.0	–	100.0
Clindamycin	100.0	100.0	0.0
Quinupristin-dalfopristin	100.0	0.0	0.0
Gentamycin	50.0	0.0	0.0
Streptomycin	25.0	0.0	0.0
Cefoxitin	–	–	–
Ciprofloxacin	50.0	50.0	–
Levofloxacin	50.0	0.0	100.0
Moxifloxacin	50.0	0.0	–
Vancomycin	0.0	0.0	0.0
Tetracycline	100.0	100.0	–
Tigecycline	0.0	0.0	0.0
Linezolid	0.0	0.0	0.0
Rifampicin	–	–	–

^**®**^Percentages = number of resistant antibiotics/total number of antibiotics tested

(–) indicate not tested

## Discussion

For the first time, we have provided more comprehensive data on surgical site infections at different levels of surgical care in Sierra Leone.

The incidence rate of surgical site infections in the two hospitals is 11.5% which is similar to the previously reported SSI incidence rates of 7.3% and 10.9% in Sierra Leone [10, 11]. Although the incidence of SSI reported in this study is similar to the WHO’s global estimates of 10.8% in low- and middle-income countries [1], the incidence of SSI reported in neighboring Nigeria and Ghana is even higher (15.6% and 32.6% respectively) [4, 15]. In our setting, it is common to use antibiotics for surgical prophylaxis [16] and one would think that the relatively low incidence of SSI can be explained by the high preoperative, intraoperative, and postoperative antibiotic use rates recorded in this study. However, according to statistical analysis, there is no significant correlation between the occurrence of SSI and the use of antibiotics. In our study, when more than two surgeons perform an operation, the patient is 70% less likely to have an infected surgical wound. Perhaps this protected some patients from acquiring an SSI, thereby reducing its incidence.

Nonetheless, similar to other studies [4, 15, 17–19], we identified several predictors of SSI, including ASA score ≥ 2, male sex, preoperative admission for more than 24 h, duration of surgery ≥ 2 h, administration of general anaesthesia, contaminated wound type, and elevated fasting blood glucose. Worthy of note is that some of these risk factors reflect a common scenario of the severe premorbid state of many patients in Sierra Leone and thus implies that early health-seeking behavior is critical to the prevention of surgical site infections. For example, a higher preoperative ASA score is a recognized predictor of poor postoperative outcomes including the occurrence of SSI [20].

In the past 10 years, there seem to be regional differences in the bacterial isolates of postoperative wounds. On the African continent, more gram-negative bacteria than gram-positive bacteria are isolated from postoperative wounds of surgical patients [21–23]. In the Middle East, *Staphylococcus aureus* is a common isolate of post-operative wounds [24, 25]. According to evidence from European countries obtained from a point prevalence survey conducted by the European Center for Disease Control and Prevention (ECDC), gram-positive cocci, including *S. aureus*, are the most commonly isolated bacterial group in postoperative wounds [26]. In our study, similar to the studies from the African region [21–23], many of the isolates from postoperative wounds of patients attending surgeries in Sierra Leone are gram-negative multi-drug resistant bacilli. There are no isolates of *S. aureus*, and the Gram-positive isolates of the postoperative wounds are *Enterococci*, *S. haemolyticus*, and a rare emerging pathogen *Kocuria Kristaenae*. The absence of *S. aureus* including the Methicillin-Resistant strain (MRSA) in post-operative wounds may be due to the limited colonization of patients and healthcare providers by these bacteria in this setting. Thus, the use of antibiotics for surgical prophylaxis, although universal in principle, must be adjusted according to the specific context, taking into account the local antibiotic use and resistance data. As such, the synthesis of local normative data should form the foundation of country-specific guidelines on surgical prophylaxis.

Moreover, the prevalence rate of the extended-spectrum beta-lactamase-producing *Enterobacteriaceae* is slightly higher than previously reported in Sierra Leone (59.8% vs. 58.0%) [27] and the African continent as a whole (59.2% vs. 28.0%) [28]. As the prevalence of carbapenem-resistant *Enterobacteriaceae* (CRE) had also increased from 1.3% in 2018 [27] to 8.4% in the present study, urgent actions are needed to set up and operationalize robust hospital-based antimicrobial stewardship programs and strengthen infection prevention and control practices. However, susceptibility to piperacillin/tazobactam, amikacin, and carbapenems was recorded in many of the gram-negative bacteria and they could therefore be recommended for empiric use in the treatment of surgical site infections in this setting where laboratory capacity on microbiology methods is limited [16]. Nonetheless, these drugs must be used with caution to prevent the development of antibiotic resistance.

Our study has limitations and strengths. First, the assessment of the risk factors of SSI is limited to patient characteristics and some healthcare delivery activities. The study did not capture data on the influence of environmental factors and hand hygiene practices of the healthcare workers in the occurrence of surgical site infections. On discharge, patients were scheduled for follow-up through telephone calls, and a small group of patients did not have telephone numbers and were therefore unreachable. Some of these patients could have acquired an SSI that was not detected by the research team thus underestimating the true burden of SSI. It is also possible that patients who developed an SSI but received postoperative care in another hospital could not provide an accurate description of their postoperative wounds. Despite these inherent challenges of a typical SSI surveillance program in a resource-limited setting, we have provided more comprehensive data on SSI at different levels of surgical care in a low-income country and highlighted the huge burden of multidrug-resistant bacteria in postoperative wounds.

## Conclusion

We found that the incidence of surgical site infections in two hospitals in Sierra Leone was comparable to other low- and middle-income countries but many of the postoperative wounds grew multidrug-resistant bacteria including ESBL-producing *Enterobacteriaceae*. Therefore, routine surveillance of surgical site infections and related antibiotic resistance is needed to guide the detection, management, and prevention in resource-limited settings.

At the same time, we highlighted several predictors of postoperative wound infections in Sierra Leone, many of which reflect that critically ill patients are receiving surgical services in these hospitals, and we thus add our voices to those who advocate for the improvement of health-seeking behaviors in low- and middle-income countries.

## Data Availability

The data supporting this article is available in the repository of University of Sierra Leone and will be made easily available on request to the corresponding author when required.

## Abbreviations

ASA: American Society of Anaesthesiologist
AST: Antibiotics Susceptibility Testing
CRE: Carbapenem resistant *Enterobacteriacea* (CRE)
ECDC: European Center for Disease Control and Prevention
ESBL: Extended-spectrum beta-lactamase (ESBL)
HAI: Healthcare associated infections
HIC: High income countries
LMIC: Low and middle income countries
SSI: Surgical site infections
WHO: World Health Organization
